# Understanding PET Hydrolysis via Reactive Molecular Dynamics Simulation and Experimental Investigation

**DOI:** 10.1021/acs.jpcb.5c03080

**Published:** 2025-06-23

**Authors:** Shuangxiu Max Ma, Patrícia Pereira, Christian W. Pester, Phillip E. Savage, Bhavik R. Bakshi, Li-Chiang Lin

**Affiliations:** † William G. Lowrie Department of Chemical and Biomolecular Engineering, 2647 The Ohio State University, Columbus, Ohio 43210, United States; ‡ Department of Chemical Engineering, The Pennsylvania State University, University Park, Pennsylvania 16802, United States; § Department of Materials Science and Engineering, 5972 University of Delaware, Newark, Delaware 19716, United States; ∥ School for Engineering of Matter, Transport and Energy, 7864 Arizona State University, Tempe, Arizona 85281, United States; ⊥ School of Sustainability, 7864 Arizona State University, Tempe, Arizona 85281, United States; # School of Complex Adaptive Systems, 7864 Arizona State University, Tempe, Arizona 85281, United States; ¶ Department of Chemical Engineering, 33561 National Taiwan University, Taipei 10617, Taiwan

## Abstract

Polyethylene terephthalate (PET), a widely used polymer in packaging applications, has posed significant environmental challenges due to its resistance to environmental degradation. Chemical recycling via hydrolysis offers a circular solution by breaking PET down into its monomers, terephthalic acid and ethylene glycol, which can then be repolymerized into new PET. Despite its promise, the detailed pathways of PET hydrolysis—particularly the interplay between hydrolysis and thermal degradation—remain a topic of scientific debate. We combine reactive molecular dynamics (MD) simulations with experimental studies to elucidate key reaction pathways, intermediate species, and the temperature-dependent evolution of degradation products. Molecular dynamics simulations offer detailed insights into molecular motions and interactions that are often elusive in experimental setups, thus enhancing our understanding of the complex dynamics at play during PET decomposition. By systematically examining bond dissociation, intermediate species, and product formation at various temperatures, this study elucidates how hydrolysis and thermal degradation pathways evolve and interact. Furthermore, a severity index approach is employed to directly compare TPA yields from simulations with corresponding experimental data.

## Graphical abstract

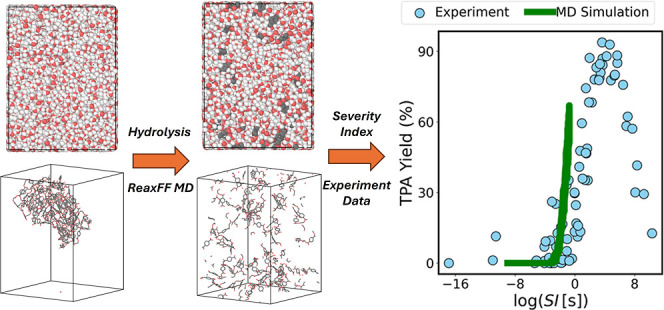

## 1 Introduction

Polyethylene terephthalate (PET) is one of the most widely used synthetic polymers, predominantly in the packaging industry for beverage bottles, food containers, and various consumer goods.^1^ Despite improved management of packaging waste, plastic bottles and other debris often end up in the environment, causing widespread harm, especially in marine ecosystems, where they degrade slowly.^2^ The persistence of PET waste and the complexities of its mechanical and chemical recycling pose significant environmental challenges.^3^ Chemical recycling of PET through hydrolysis is considered a promising method, as it decomposes the polymer into its monomers—terephthalic acid (TPA) and ethylene glycol (EG)—which can then be repolymerized into virgin PET.^4–6^

The degradation pathways of PET are complex.^7–9^ Both neutral and catalyzed hydrolysis of PET yield byproducts, such as mono-(2-hydroxyethyl)­terephthalic acid (MHET), bis­(2-hydroxyethyl) terephthalate (BHET), benzoic acid (BA), and isophthalic acid (IPA).^8,10–12^ While previous studies have shed light on the complex reaction pathways, which potentially yield products such as MHET, BHET, EG, and TPA in specific proportions, many aspects of PET hydrolysis remain unclear.^7,8,13–16^ These include whether BHET forms solely from PET or whether there are secondary reactions involving EG formed in situ, and which pathways lead to secondary products such as IPA or gaseous species.

Traditional experimental approaches provide macroscopic observations of PET hydrolysis but lack the resolution to capture molecular–scale interactions and transient species. In contrast, molecular dynamics (MD) simulations enable the direct observation of bond dissociation events, intermediate formation, and reaction pathways at the atomic level. Despite this advantage, MD simulations require experimental validation to ensure their relevance to real–world reaction conditions. By combining experimental results with reactive MD simulations using the reactive force field (ReaxFF) developed by van Duin et al.,^17^ this study bridges the gap between fundamental reaction modeling and practical processes.

ReaxFF employs bond-order-based parameters trained against density functional theory (DFT) calculations, enabling the accurate simulation of chemical reactions in complex systems. This enables the simulation of large systems with high precision. The application of ReaxFF for the study of polymers has shown significant potential for advancing our understanding of macromolecular behavior in complex environments, offering a robust framework for modeling chemical reactions and mechanical interactions at the molecular level.^18,19^ As examples, Panczyk et al.^20^ investigated the surface chemistry of degraded PET, demonstrating that key material properties, including density, Young’s modulus, and Poisson’s ratio, can be accurately predicted, and offering valuable insights into the stability and environmental behavior of PET. Ma et al.^21^ also employed ReaxFF to elucidate the reaction network and perform kinetic modeling of PET thermal cracking and reforming, showcasing its ability to capture detailed chemical pathways with strong agreement to existing experimental findings. Fayon et al.^22^ conducted molecular simulations to evaluate the interfacial interactions and adhesion properties of PET surfaces in various base/alcohol systems, providing insights into molecular-level phenomena governing PET degradation.

This dual approach served several key objectives. The first was to determine whether hydrolysis follows a direct monomeric release mechanism or involves secondary transformations. A second objective was to quantify the impact of temperature and pressure on PET degradation, thereby distinguishing hydrolysis-dominant conditions from thermally accelerated pathways. In addition, we aimed to investigate how water molecules participate in bond cleavage, with experiments confirming these effects under industrially relevant conditions. Finally, we analyzed the interplay between hydrolysis and pyrolysis pathways to identify the conditions under which PET degradation shifts from monomer recovery to uncontrolled fragmentation.

## 2 Methodology

### 2.1 Computational Details

Reactive MD simulations were performed to study the PET-water system. A PET polymer was constructed with 100 ethylene terephthalate units corresponding to the molecular weight of 19,200 g/mol to represent the typical molecular weight of the PET polymer for simulation. This is close to the range of experimental PET, as virgin and waste PET presents *M*_n_ of around 20,000–40,000 g/mol.^23^ Figure 1 depicts the PET model studied in this work. The box initially had a large dimension of 60 × 60 × 80 Å^3^ to avoid overlaps of molecules. A PET/water mass ratio of 0.26 was chosen as it is similar to values used experimentally, and it provides a system that is computationally feasible to simulate. MD simulation was first conducted to refine the initial structure in the isothermal–isobaric ensemble (i.e., *NPT* ensemble (constant number of particles, temperature, and pressure ensemble) with pressure = 1 atm and temperature = 300 K); the simulation compressed the structure to reach the final density of 0.99 g/cm^3^ to represent the PET with water environment. The simulations of PET degradation were then performed under the canonical ensemble (i.e., *NVT* ensemble (constant number of particles, volume, and temperature)) with a Nosé–Hoover thermostat to simulate PET hydrolysis. For simulating hydrolysis, the temperature of the systems was first ramped up to the desired temperature at a heating rate of 100 K/ps, followed by holding at the target temperature for 1 ns. A time step of 0.1 fs and a temperature damping constant of 100 fs were used in all ReaxFF MD simulations. To capture bond-breaking within such a rather short time scale, simulations at 1400–2000 K were conducted, compressing minutes of practical-temperature chemistry into nanoseconds. With their product distributions matching experimental trends, the elevated-temperature simulations therefore yield statistically robust coverage of the reaction network while remaining faithful to actual hydrolysis chemistry. For each system, five independent simulations were conducted with distinct velocity seeds, and all kinetic quantities and yields reported herein are the average values. In addition, to investigate the influence of pressure on the system while ensuring the simulation settings mimic those of a sealed reactor, the density was adjusted by compressing the periodic simulation box to the targeted density before initiating the heating process.

**Figure 1:**
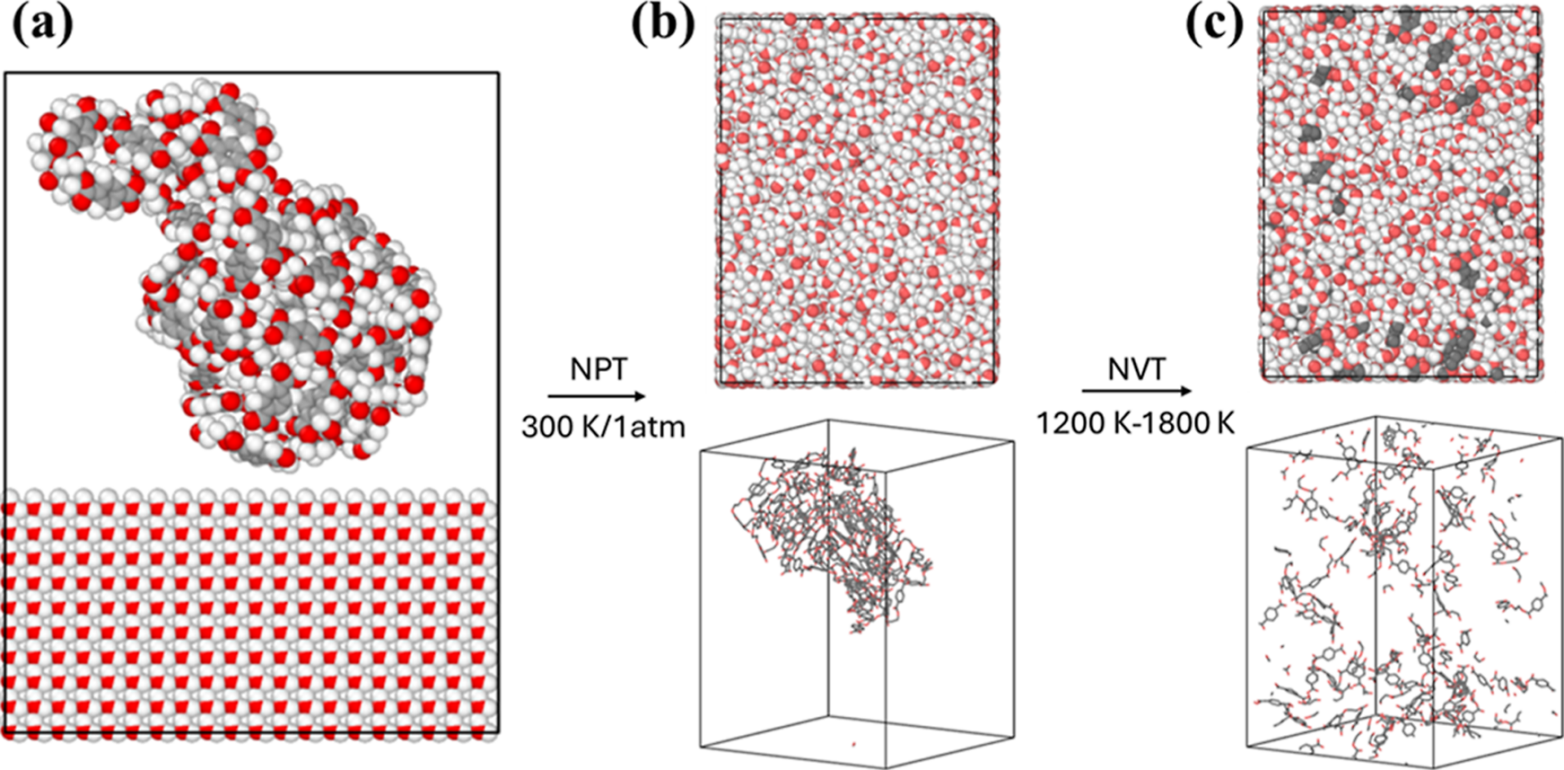
Illustration of the procedure to build the simulated PET-water system: (a) Building a long PET chain (i.e., 100 monomers as shown) with water molecules, and the molecule is placed in a large periodic domain. (b) The system was first compressed by a simulation under the *NPT* ensemble (300 K and 1 atm). (c) Simulation under the *NVT* ensemble at the targeted temperature.

Molecular simulations require reliable descriptions of inter- and intramolecular interactions. Reactive force fields^24^ used in this study were developed and parametrized based on quantum mechanical (QM) data,^17,24,25^ enabling them to accurately describe bond association and dissociation in reactive systems while maintaining computational efficiency. Such force fields are based on bond orders determined by the distance between pairs of atoms. Various energy contributions can then be accordingly computed, including bond energy (*E*_bond_), overcoordination energy (*E*_over_), under-coordination energy (*E*_under_), valence angle energy (*E*_val_), penalty energy (*E*_pen_), torsion energy (*E*_tors_), conjugation energy (*E*_conj_), and nonbond van der Waals (*E*_vdWaals_) and Coulomb (*E*_Coulomb_) interactions. In this work, the force field developed by van Duin et al.^26^ for the hydrocarbons/water systems was used to describe the PET hydrolysis. This force field has also been successfully utilized in previous MD studies, which accurately describe the interaction of hydrocarbon/water molecules in the condensed phase.^27–29^ Further validations against experimental data were also conducted with results discussed in a later section to verify its suitability in describing the system of interest in this study. Hydrogen and oxygen atoms from water were labeled as *H*_w_ and *O*_w_, respectively, to distinguish them from H and O atoms in other molecules.

### 2.2 Experimental Methods

We used an experimental data set for the hydrolysis of postconsumer PET water bottles and terephthalic acid (TPA) decarboxylation and esterification, which can be found in Pereira et al.^7,10,11^ Briefly, PET pellets were placed in a 4 mL stainless steel reactor with a 1/10 mass ratio of feed to water. The reaction was conducted in a sand bath maintained at the desired temperature and duration. Then, the reactors were immediately quenched in room temperature water to stabilize their contents before opening. Upon opening, the contents were filtered with aliquots of 10 mL of water to separate the liquid and solid phases. The liquid phase was evaporated, and the solid residue was dissolved in dimethyl sulfoxide (DMSO; from Millipore Sigma) for subsequent quantification by High-Performance Liquid Chromatography (HPLC). Mass yields were calculated as the mass of the product divided by the maximum amount of product to be produced from the initial mass of the PET repeating unit.^12^

## 3 Results and Discussion

In this section, the activation energy is simulated and compared against experimental values. Chain breaking process and commonly key species are then discussed to offer an atomistic overview of the PET-water system, followed by discussions on the use of the severity index.

### 3.1 Activation Energies

The activation energy (*E*_a_) for the PET hydrolysis determined through a series of MD simulations was directly compared with experimental data^10^ to assess the accuracy of the MD simulations, particularly the chosen force field. In these simulations, the temperature of the PET system was systematically increased to 1400 at 100 K/ps and maintained at each temperature (from 1400 to 2000 K) for 20 ps. This brief isothermal reaction period was designed to simulate conditions dominated by hydrolytic reactions, while thermal reactions, though possible, were presumed to proceed at a much slower rate under these conditions. The reaction rate constants were calculated based on the reduction in mass of species containing over 20 carbon atoms (the number in a PET dimer as the smallest, purely condensed-phase fragment that still behaves as polymeric solid) during hydrolysis at each isothermal temperature for capturing the chain cleavage process we tend to isolate. The logarithm of these reaction rate constants (ln­(*k*)) was plotted against the reciprocal of the temperature (1/*T*) in an Arrhenius plot as shown in Figure 2. The activation energy (*E*_a_) from the MD simulations was determined to be *E*_a_ = 104 kJ/mol which aligns well with experimental data,^10^ that presented a range of 90–123 kJ/mol.^30^ The activation energy in the simulations was determined from the initial stage of hydrolysis, corresponding to a conversion below 2.5%. By focusing on this low-conversion range, which is unswollen and diffusion-free, we ensure that the rate constant primarily reflects hydrolysis rather than competing thermal pathways. Extrapolating the line for the data from the MD simulation to lower temperatures shows it accurately predicts the experimental data set^10,31–35^ on PET hydrolysis across different studies. This strong correlation between simulation and experimental results supports the validity of the potential model used in the MD simulations.

**Figure 2:**
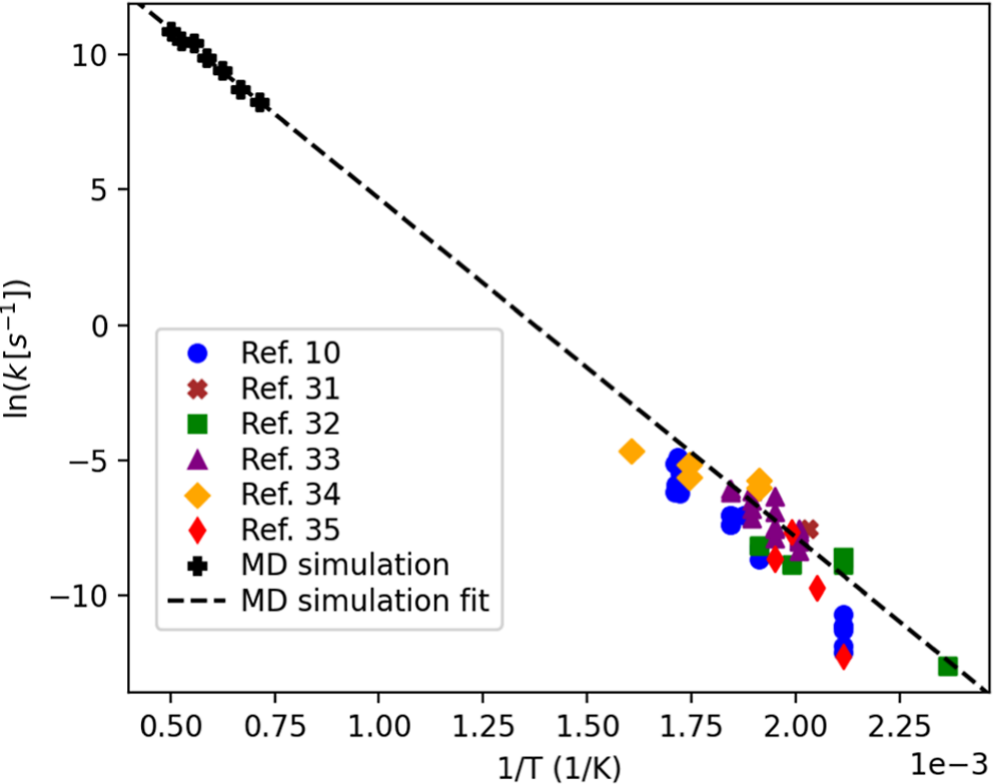
Arrhenius plot for MD-simulated PET neutral hydrolysis (kinetic constants *k* represent the PET conversion) and the extrapolated results from MD simulation (i.e., black dashed line) and experimental data.

### 3.2 Atomistic Overview of the PET Hydrolysis

With the trajectories collected from MD simulations, the changes in the length of the longest PET chain were analyzed to gain insights into the chain-breaking process during hydrolysis. As shown in Figure 3a, at 1400 K, the PET chains tend to maintain the initial structure of 100 monomers for a longer time than at higher temperatures. As the temperature rises to 1600 K, 1800 K, and finally 2000 K, there is a significant decrease in the length of the longest chain at a given time, with the majority of the chains breaking down into much shorter fragments. The change for the longest, second-longest, and third-longest chains can be found in Figure S1a, and the influence of the temperature on the chain length change can be seen in Figure S1b. The experimental data (Figure 3b) also show that the yields of undissolved solids (unreacted PET) decrease with increasing temperature and reaction times.

**Figure 3:**
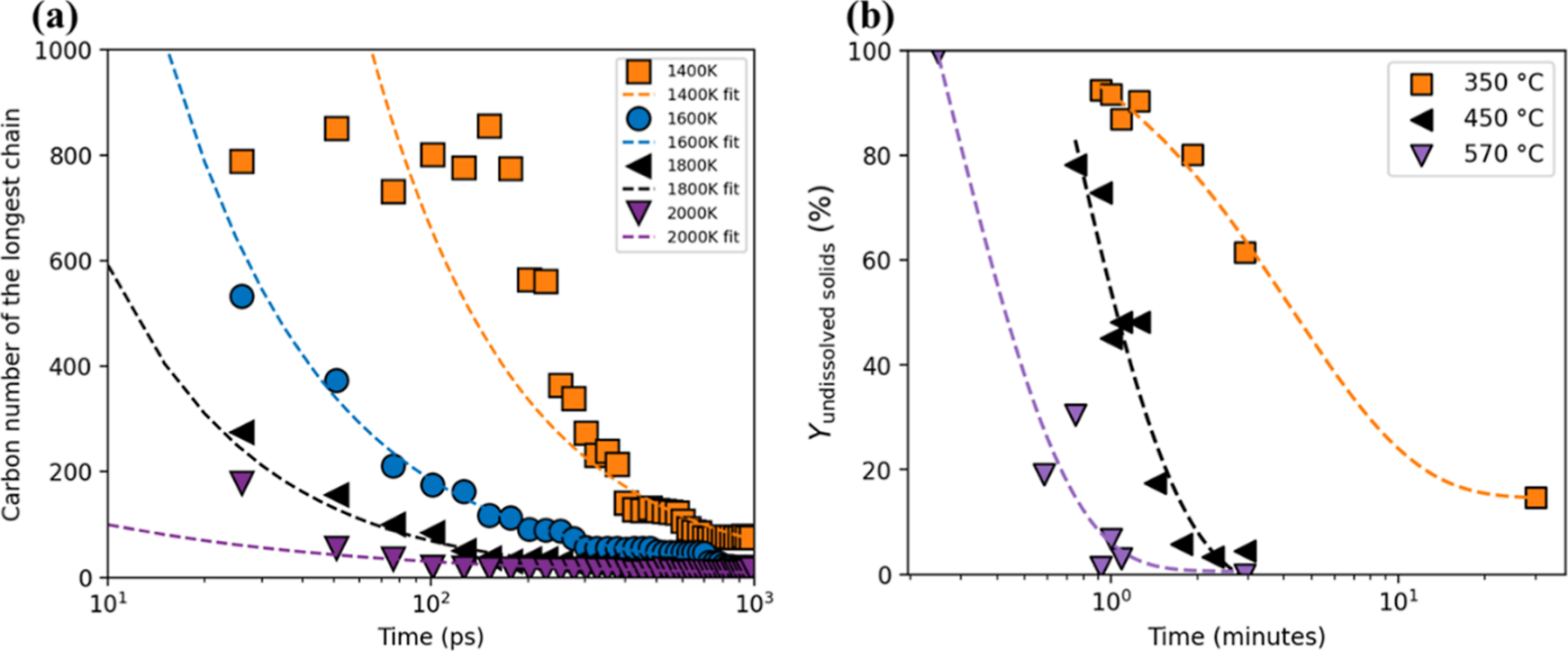
(a) Carbon number of the average longest PET chain during hydrolysis at various temperatures from simulation (data points plotted every 25 ps for clarity) with power-law fit. (b) Temporal variation of the yield of undissolved solids with power-law fit (inclusive of unconverted PET and oligomers). Data in Figure 3b is adopted from Pereira et al.^11^ (Royal Society of Chemistry (CC BY-NC 3.0)).

### 3.3 Analysis of Water-Related Species from MD Simulations

Water-related species are analyzed to further understand the reaction process, during hydrolysis at 1800 K, Figure 4a shows a gradual decline in the total number of H_2_O molecules over the first 200 ps, signifying net water consumption attributable to hydrolytic reactions. Beyond this time frame, the water content appears to stabilize, suggesting other types of reactions (e.g., thermal) dominate. Simultaneously, as illustrated in Figure 4b, the OH^•^ radical concentration initially increases alongside active hydrolysis—mirroring the drop in water content—until about 200 ps. Beyond that point, water consumption levels off, suggesting that hydrolysis is no longer the primary pathway. Consequently, the dominant mechanism shifts toward thermal degradation. The populations of H^+^, OH^–^, and H_3_O^+^ fluctuate (Figure 4c), reflecting cyclical proton-transfer events in which these ionic intermediates are generated, consumed, and regenerated.

**Figure 4:**
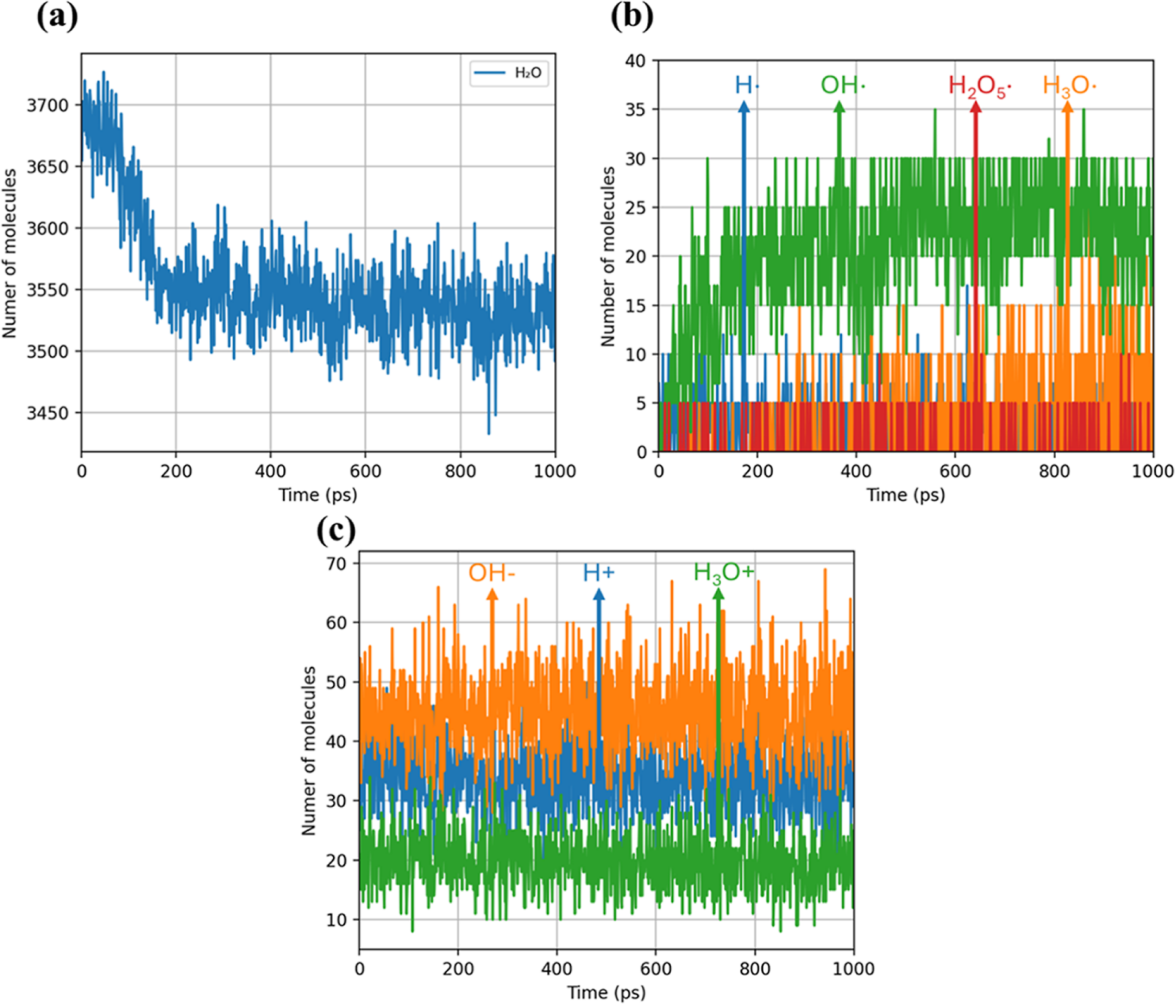
Time evolution of water-derived ionic and radical species during PET hydrolysis in MD simulations at 1800 K: (a) water molecules, (b) Populations of key radical species (H^•^, H_3_O^•^, HO^•^, and H_2_O–H_3_O^•^) and (c) ions (H^+^, OH^–^, and H_3_O^+^) over the course of the reaction.

### 3.4 Analysis of Species from MD Simulations

To further illustrate the temporal evolution of products and byproducts within different carbon number ranges from PET hydrolysis via MD simulation, results at 1800 K are shown in Figure 5. This elevated temperature accelerates reactions within the 1 ns simulation window, showing complete trends of the change of species over time. Figure 5a clearly shows that molecules with larger numbers of carbon atoms are progressively broken into shorter fragments. By the end of the simulation period, most of the initial polymer chain was reduced to small oligomers or monomers that have carbon numbers under 10. Figure 5b,c provide a detailed view of the formation and evolution of small carbon-chain fragments. Compared with C8, C2, and C1 species, C10 fragments appear in much lower concentrations. This observation suggests that C10 fragments are less stable and more susceptible to further decomposition into smaller units. Such fragments can be MHET from TPA esterification with EG.^7^ The persistence of C8 species (e.g., TPA) throughout the reaction indicates that these are key aromatic products and byproducts in the degradation pathway. Among species with lower carbon number, C2 (e.g., EG) and C1 are identified as the major products. Initially, the concentration of C2 species increases rapidly as the PET chains begin to break down, indicating that C2 is produced during the early stages of hydrolysis, likely as a direct result of the cleavage of ester linkages within the PET polymer (i.e., resulting in EG). However, as the reaction progresses, there is a notable decrease in the concentration of C2 species, which coincides with a simultaneous increase in C1 species. This trend suggests a secondary transformation process, where C2 fragments are further broken down into smaller C1 units. In addition, we note that Sato et al.^34^ experimentally observed a decrease in EG with increasing times at *T* > 300 °C.

**Figure 5:**
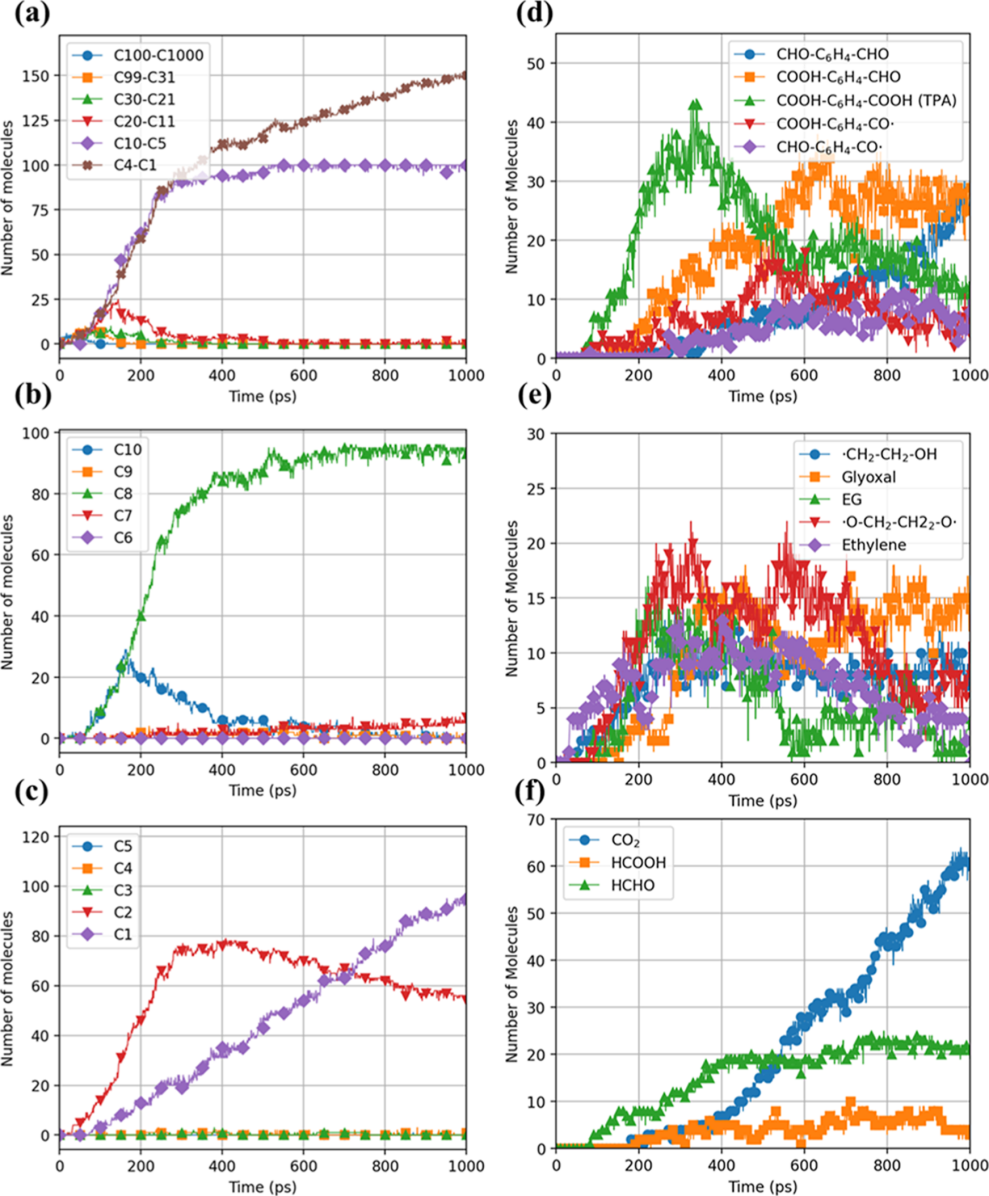
Time evolution of molecular species during PET hydrolysis observed in MD simulations at 1800 K. (a) Distribution of chain lengths over time, indicating progressive chain scission. Formation of carbon-chain fragments of (b) C6–C10 and (c) C1–C5. Formation of (d) various aromatic compounds, including terephthalic acid (TPA), (e) degradation products such as glyoxal and ethylene glycol (EG), and (f) various light gas compounds.

The persistence of species, including C10, C8, C2, and C1, throughout the reaction suggests that they are key products and byproducts in the degradation pathway. Further details are provided in Figure 5d, which illustrates the formation and consumption of C8 species during PET hydrolysis, with a focus on terephthalic acid (TPA) and its subsequent reactions. Initially, TPA was produced as a significant product of the hydrolysis process. However, its concentration decreases over time, indicating that TPA is not a terminal product but undergoes further reactions under the studied conditions. The accompanying reaction pathway highlights the role of water in facilitating these transformations, particularly in the reduction of TPA and the production of species containing aldehyde (−CHO) groups, such as COOH–C_6_H_4_–CHO (4-formylbenzoic acid) and CHO–C_6_H_4_–CHO (terephthalaldehyde). However, note that under experimental conditions at much lower temperatures, such reactions typically involve the use of strong reducing agents (e.g., lithium aluminum hydride) or enzymes.^36^ Therefore, even though these species are observed in MD simulations, we do not expect to find them in experimental operating conditions using water alone.

It is important to note that other C8 species, such as isophthalic acid (IPA), can arise as minor derivative isomers of TPA.^7,8^ IPA was observed in small quantities (<0.4% IPA yield) for a system with pure TPA powder and water from isothermal hydrolysis at 270–308 °C for 30 min.^7^ This indicates that there are potentially two routes for IPA appearance after PET hydrolysis: (1) direct release from IPA trapped in PET repeating units, and (2) from isomerization of TPA. However, route 1 seems to produce the majority of IPA after PET hydrolysis, as on average, carbonated drink bottles and PET films contain 2% IPA.^37^ Prior research also suggested that IPA yield peaks and then declines at higher temperatures, indicating potential decomposition similar to TPA.^8^ Additionally, TPA was observed to participate in side reactions where it produced a mixed ester of TPA with ethylene glycol, diethylene glycol, and triethylene glycol ester, which were experimentally observed.^38^

Figure 5e reveals the complexity of the C2 products formed during thermal treatment and that at higher temperatures, EG is unstable and can be further decomposed into smaller molecules such as glyoxal and other gaseous radical byproducts. The instability of EG at elevated temperatures emphasizes the dynamic nature of the hydrolysis process, where intermediate products continue to break down into simpler and more volatile compounds. Previous studies showed experimentally as well that EG from PET hydrolysis decreases with increasing temperature and reaction time,^39^ via dehydration and dimerization, leading to the formation of acetaldehyde, diethylene glycol, and triethylene glycol (molecules not observed through MD simulation because of the high simulated temperatures).^8,34^ Figure 5f also shows that CO_2_ is the predominant terminal gas species, but other C1 species such as formaldehyde (HCHO) and formic acid (HCOOH) are also formed, likely due to the participation of water molecules in the reactions.

Figure 6 depicts the time evolution of molecular species observed during PET hydrolysis at 1800 K in MD simulations, focusing on benzoic acid and BHET (bis­(2-hydroxyethyl) terephthalate). Figure 6a shows that benzoic acid begins to form after approximately 400 ps, with its concentration fluctuating over time, indicating transient stability and potential further degradation into smaller species. This late emergence suggests that benzoic acid is a secondary product of initial hydrolysis or thermal degradation. Figure 6b reveals that BHET forms much earlier, peaking around 200 ps, and then rapidly decreases, suggesting that BHET acts as an intermediate, breaking down into smaller products such as terephthalic acid (TPA) and ethylene glycol (EG). These trends align with the previous analysis of reaction pathways, which identifies BHET as a key intermediate that undergoes further hydrolysis. The differences in formation timelines for benzoic acid and BHET emphasize the sequential and dynamic nature of PET hydrolysis, where intermediates like BHET are quickly consumed while secondary products like benzoic acid accumulate over time.

**Figure 6:**
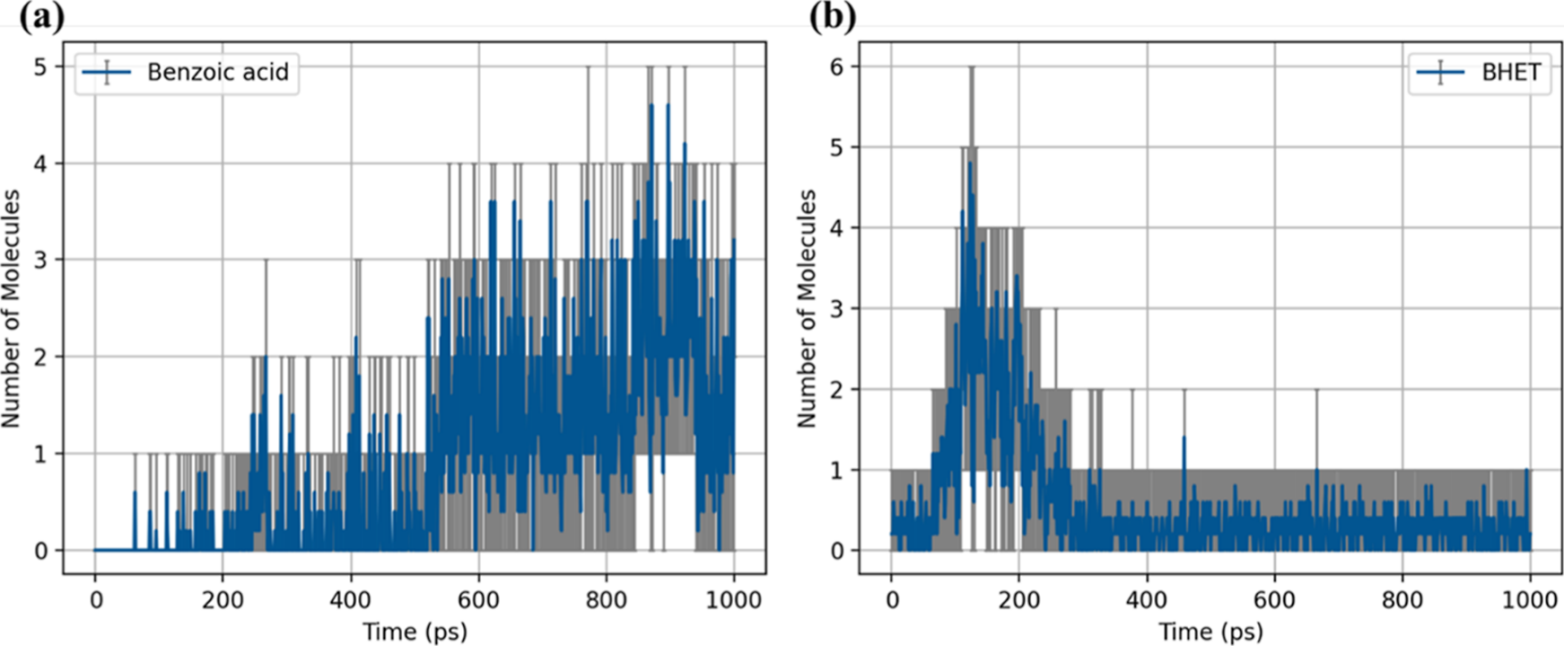
Time evolution of molecular species during PET hydrolysis observed in MD simulations at 1800 K: (a) benzoic acid and (b) BHET (bis­(2-hydroxyethyl) terephthalate). The blue line represents the average observed number of five independent runs with the error bar in gray.

### 3.5 Reaction Pathways

Figure 7 shows a reaction network for PET degradation, illustrating the dual processes of hydrolysis and thermal degradation. This network was constructed by comparing the SMILES-based product lists generated at each MD simulation time step and applying chemical reasoning about the most likely pathways for all the simulations from 1400 to 2000 K. The network highlights how PET breaks down into key products like TPA and EG, as well as smaller, more reactive species when exposed to elevated temperatures. In addition, details of the decomposition reaction of TPA are discussed, as higher reaction temperatures render TPA reactive; omitting its decarboxylation and dehydration would exaggerate the isolated-TPA yields and skew the gas evolution. Capturing these secondary pathways is essential to provide a comprehensive landscape of reactions that are beyond primary ester-bond hydrolysis. Notably, the pathways are color-coded to distinguish between radical reactions (red) and molecular processes (blue), thereby underscoring how the presence or absence of radical intermediates can alter the degradation routes under different conditions. The reaction network is not meant to be comprehensive (e.g., not every coreactant or coproduct is shown for every reaction) but rather illustrative of the major pathways connecting the major products. It should be noted that although the simulation performed herein was at a constant volume, both the MD and experimental studies suggest that the final product distribution and the reaction network are independent of pressure (see Figure S2). Therefore, employing such simulation conditions should not alter the key reaction pathways.

**Figure 7:**
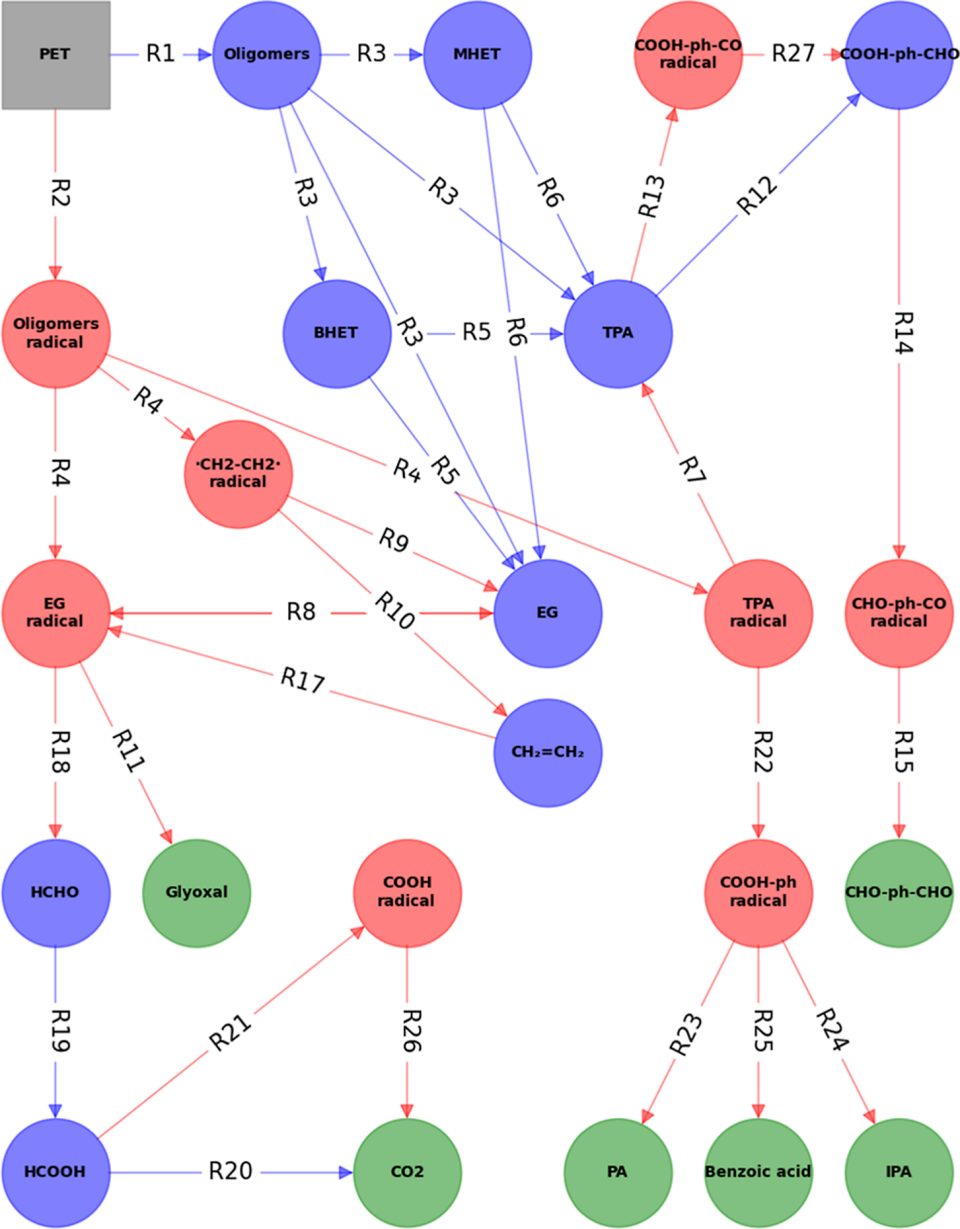
Schematic diagram of key reactions observed from atomistic MD simulations. Radical species and related reactions are marked red, and the ending nodes are marked green. (–ph– represents a phenyl moiety). The network is a unified map derived by pooling elementary events that appeared in any of the four isothermal ensembles (1400 K, 1600 K, 1800 K, 2000 K).

At the core of the reaction network, the hydrolysis process is depicted as the primary pathway. In this pathway diagram, water molecules interact with PET polymer chains, cleaving them into smaller oligomers. These oligomers undergo further hydrolysis, leading to the formation of monomers such as MHET, BHET, TPA, and EG. These reactions (R1, R3, R5, and R6) are characteristic of the hydrolytic breakdown of PET, which tends to dominate under lower temperatures, favoring the production of end products like TPA and EG. However, as temperature increases, the reaction network expands to include pyrolytic mechanisms, which significantly alter the degradation landscape. Under these conditions, thermal energy induces the formation of radical species such as diradical ethylene (^•^CH_2_–CH_2_^•^) and glyoxal, which are generated through thermal cracking and subsequently participate in a series of secondary reactions (R4, R10, R11, and others). These reactions highlight the formation of highly reactive intermediates that are less prevalent under purely hydrolytic conditions. For instance, the network shows how EG radicals can decompose into glyoxal (R11) and formaldehyde (R18), illustrating the transition from monomeric products to more reactive, smaller species as the reaction conditions shift from hydrolysis-dominated to pyrolysis-dominated. Also note that BHET and MHET are primarily formed from PET depolymerization, as reaction with EG is rare for their production. Furthermore, TPA is observed to react with oligomers, though the extent of autocatalysis is unclear.

The network also shows the interplay between these radical species and other products of degradation. Reactions such as the transformation of TPA into aldehyde and ketone derivatives (R12, R13, R14, R15) underscore the complexity of the pyrolytic pathway, where a multitude of byproducts are formed through a series of oxidation and reduction steps. Additionally, the formation of carbon dioxide (CO_2_) through the sequential breakdown of formic acid (HCOOH) (R19, R20) represents the final stage of radical-driven degradation, emphasizing the extensive chain of reactions that can occur when thermal energy is introduced and the reaction path of producing CO_2_ and H_2_ is favored in the presence of water. Comparing this reaction network to experimental data reveals that, while hydrolysis remains the primary mechanism at lower temperatures (i.e., experimental conditions), the inclusion of pyrolysis in simulations leads to a broader and more complex array of reaction pathways and intermediates. The simulation data indicates a more diverse range of products at higher temperatures, primarily due to pyrolytic mechanisms. As temperature increases (red arrow), thermal processes dominate, while at lower temperatures (blue arrow), hydrolytic pathways prevail. Overall, this highlights the importance of temperature and reaction conditions in determining the dominant degradation pathways and the resultant products.

### 3.6 Comparison of Severity Index

To make a direct comparison between MD simulation results and the experimental PET hydrolysis data, we use the severity index (SI) as per Overend and Chornet.^40^ SI combines temperature and time effects into a single metric. Operationally, SI weights every moment of a reaction by its thermal driving force above a reference temperature and sums those weighted exposures over the total reaction time, so that two experiments (or simulations) with the same SI deliver the same cumulative extent of heat input regardless of the specific temperature–time combination.

For an isothermal reaction, SI is simply the product of a rate constant (*k*) and batch holding time (*t*). If the reaction is nonisothermal, as in many of our experimental studies, one needs to integrate as shown in eq 1. Since the present study includes parallel thermal degradation (td) and hydrolysis (hyd) reaction paths, the sum of the rate constants for the two paths is used.

1 $$SI=\int_{0}^{t}\left(e^{-E_{a,hyd}/R\left(\frac{1}{T}-\frac{1}{T_{ref}}\right)}+e^{-E_{a,td}/R\left(\frac{1}{T}-\frac{1}{T_{ref}}\right)}\right)dt$$

*E*_a_ is an activation energy, *T*_ref_ is the reference temperature (700 K), *R* is the gas constant, and *t* is the batch holding time in minutes. For the hydrolysis portion of the SI, we use the same parameters as in prior work.^11^ For the thermal portion, we use parameters from experiments on PET pyrolysis^41^ (*E*_a_ = 212 kJ/mol).

Figure 8a, b show that the simulation curve at 1400 K aligns closely with the experimental data. This congruence suggests that the model accurately captures the experimental behavior under these specific conditions. Furthermore, the upward trajectory of the 1400 K line as it approaches and aligns with the high experimental yields implies that, given extended reaction durations and/or smaller MD simulation temperatures, the TPA yield might consistently reach or even exceed 80%. A closer inspection of Figure 8a reveals that the experimental TPA yield rises to a maximum at intermediate severity and then declines with higher SI. This nonmonotonic behavior is captured by the 1600 K simulation (yellow symbols), which peaks near 40–50% TPA at SI ≈ 0 and falls to ∼20% at larger SI as secondary decarboxylation of TPA overtakes its formation. For the 1400 K series (green symbols), while the same rollover is expected, it does not emerge within the 5 ns trajectories. This is attributed to time-restricted sampling (i.e., only a few nanoseconds) rather than a model limitation; the underlying mechanism is the same (i.e., side-reaction crossover) that produces the downturn as computationally observed at 1600 K and in the experiments. Nonetheless, while the computational cost of simulations limits the exploration of lower temperatures, with the severity index, the results of MD and experiments can be compared on the same scale. Above 1400 K, there are noticeable deviations between simulation and experimental data, presenting a decrease in TPA yield at lower SI. This discrepancy indicates that, at the elevated temperatures employed in the MD simulations, additional reaction pathways may be more dominant, such as the dominance of thermal degradation. This highlights the importance of maintaining temperature conditions optimized for hydrolysis to maximize TPA yield while minimizing thermal degradation side reactions.

**Figure 8:**
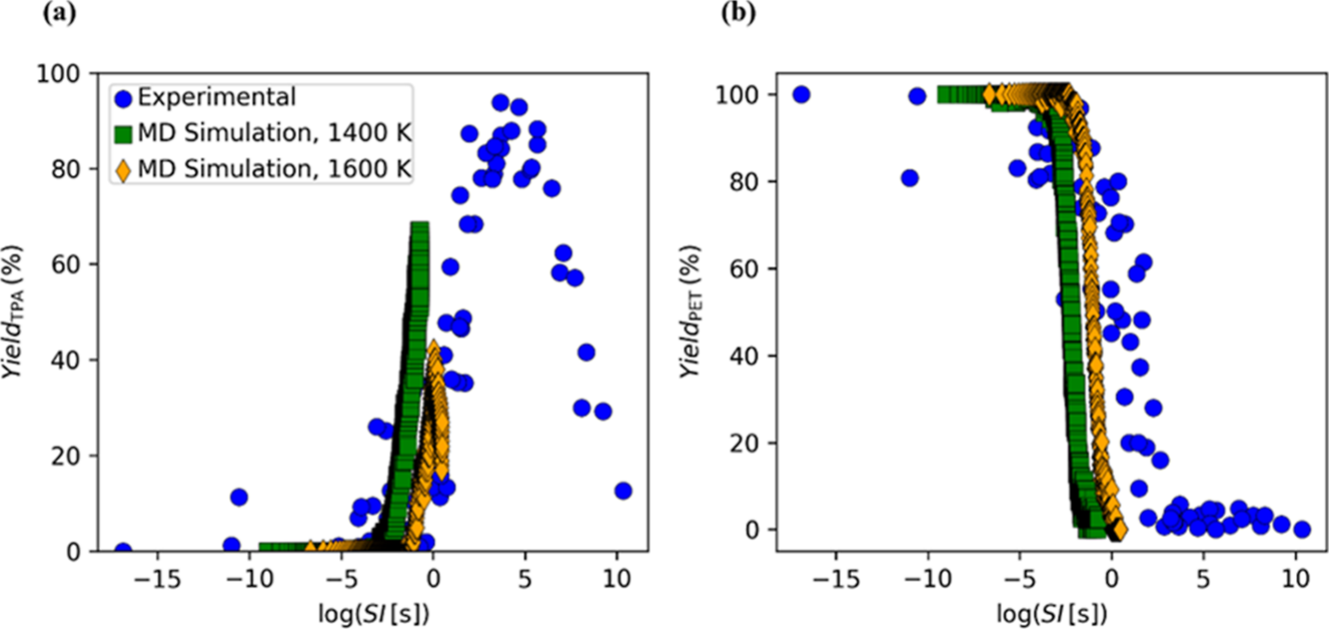
Comparison between MD simulation at 1400 and 1600 K, and experimental data points to (a) TPA yield and (b) PET yield from PET neutral hydrolysis in relation to severity index (SI).

## 4 Conclusion

This study integrates reactive molecular dynamics (MD) simulations and experiments to investigate polyethylene terephthalate (PET) hydrolysis and thermal degradation, bridging molecular insights with macroscopic observations. The formation of key degradation products was observed from MD simulation, showing consistency with experimental results. The array of byproducts identified in both the simulations and the experiments underscores the complex nature of PET decomposition under elevated temperatures. Notably, hydrolysis dominates at lower temperatures, producing terephthalic acid (TPA) and ethylene glycol (EG) as the principal products, while elevated temperatures enable additional reaction pathways through thermal degradation, leading to aromatic byproducts—such as isophthalic acid (IPA) and phthalic acid (PA)—as well as gaseous species like CO_2_.

Furthermore, both simulations and experiments capture the persistence and transformation of intermediate compounds, including bis­(2-hydroxyethyl) terephthalate (BHET) and mono-(2-hydroxyethyl) terephthalate (MHET). The severity index (SI) showed that MD simulations at 1400 K can model experimental results from hydrolysis reactions. However, this metric failed at higher MD simulation temperatures, likely because other pathways become more prevalent, highlighting the importance of carefully selecting and interpreting simulation temperatures. Overall, these findings reaffirm the complementary strengths of reactive MD simulations and experiments in elucidating PET degradation mechanisms. In summary, this integrated approach not only deepens our mechanistic understanding of PET decomposition but also provides a promising framework for optimizing and designing more sustainable processes.

## Supplementary Material



## Notes

Supplementary figures and discussion showing (i) chain-length distributions and rank-ordered breakdown of PET during hydrolysis at 1400–2000 K and (ii) pressure-effect analysis via species-evolution plots at two densities, plus the associated reference list (PDF)
