# Supplementary material for: Understanding PET Hydrolysis via Reactive Molecular Dynamics Simulation and Experimental Investigation
Source: J Phys Chem B. 2025 Jun 23;129(26):6594–603. doi: 10.1021/acs.jpcb.5c03080 (PMC12235640; doi:10.1021/acs.jpcb.5c03080)
Supplement: Supplementary file 1 [file jp5c03080_si_001.pdf]

Supporting Information

For

Understanding PET Hydrolysis via Reactive Molecular Dynamics Simulation and Experimental Investigation

Shuangxiu Max Ma[a,¶], Patrícia Pereira[b,¶], Christian W. Pester [c,*], Phillip E. Savage[b,*], Bhavik R Bakshi[d,e,f*], Li-Chiang Lin[a,g,*]

[a] William G. Lowrie Department of Chemical and Biomolecular Engineering, The Ohio State University, Columbus, Ohio 43210, United States

[b] Department of Chemical Engineering, The Pennsylvania State University, University Park, PA 16802, United States

[c] Department of Materials Science and Engineering, University of Delaware, Newark, DE 19716, United States

[d] School for Engineering of Matter, Transport and Energy, Arizona State University, Tempe, AZ 85281, United States

[e] School of Sustainability, Arizona State University, Tempe, AZ 85281, United States

[f] School for Complex Adaptive Systems, Arizona State University, Tempe, AZ 85281, United States

[g] Department of Chemical Engineering, National Taiwan University, Taipei 10617, Taiwan

[¶] These authors contributed equally to this work.

*Corresponding authors: C.W.P. (pester@udel.edu), P.E.S. (psavage@psu.edu), B.R.B. (bhavik.bakshi@asu.edu) and L.-C.L. (lclin@ntu.edu.tw)

Contents

1. Change of chain length 3

2. Pressure effect 4

3. References.. 5

1. Change of chain length

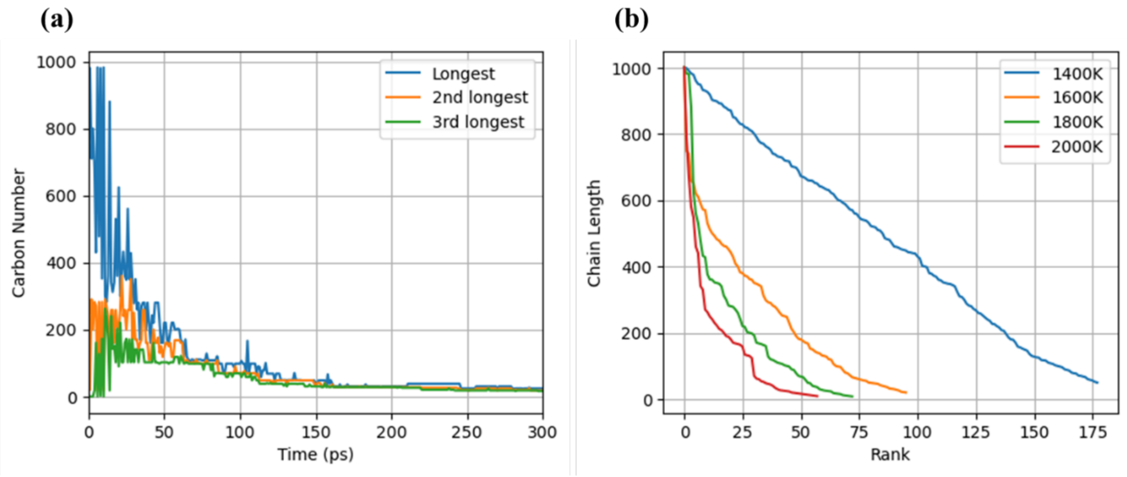

Figure S1. (a) Chain length distribution of PET during hydrolysis at 1800 K. **(b)** Chain length rank of PET during hydrolysis at temperatures from 1400 K to 1800 K.

Figure S1 provides a detailed depiction of the evolution of PET chain lengths during hydrolysis at high temperatures, with data from molecular dynamics (MD) simulations. **Figure S1a** demonstrates the rapid breakdown of the longest PET chains at 1800 K, as the carbon number of the three longest chains decreases significantly within the first 200 picoseconds (ps) of the simulation. This rapid chain scission reflects the high reactivity of PET at elevated temperatures, where hydrolysis reactions are accelerated. Beyond 200 ps, chain lengths stabilize, indicating that most hydrolysis events are complete, leaving smaller oligomers and monomers. **Figure S1b** further explores the rank-order distribution of chain lengths at various temperatures (1400 K, 1600 K, 1800 K, and 2000 K). Chain length rank is defined by ordering polymer chains from longest (rank 1) to shortest (rank n), where n is the total number of chains present at a given time point. At lower temperatures (*e.g.*, 1400 K), longer chains persist, showing slower degradation rates, while at higher temperatures (*e.g.*, 2000 K), the chain lengths are reduced drastically, with even the longest chains fragmenting into short lengths rapidly.

2. Pressure effect

Note that during MD simulations, pressure changed since the volume was kept constant (*e.g.*, for 1800 K, pressure changed from 1.8 to 2.96 GPa in **Figure S2**). We do not expect the system pressure to influence the reaction, as experiments in liquid water showed TPA yields remained statistically consistent whether the reaction was carried out at 1.5 MPa or 33 MPa at 200 ℃.[1] TPA yields were also unaffected by the state of water, whether it was saturated liquid, vapor, compressed liquid, or superheated vapor.[1] PET conversion for 100 to 250 ℃ was also not influenced by pressure from 3.4 to 5.5 MPa.[2]

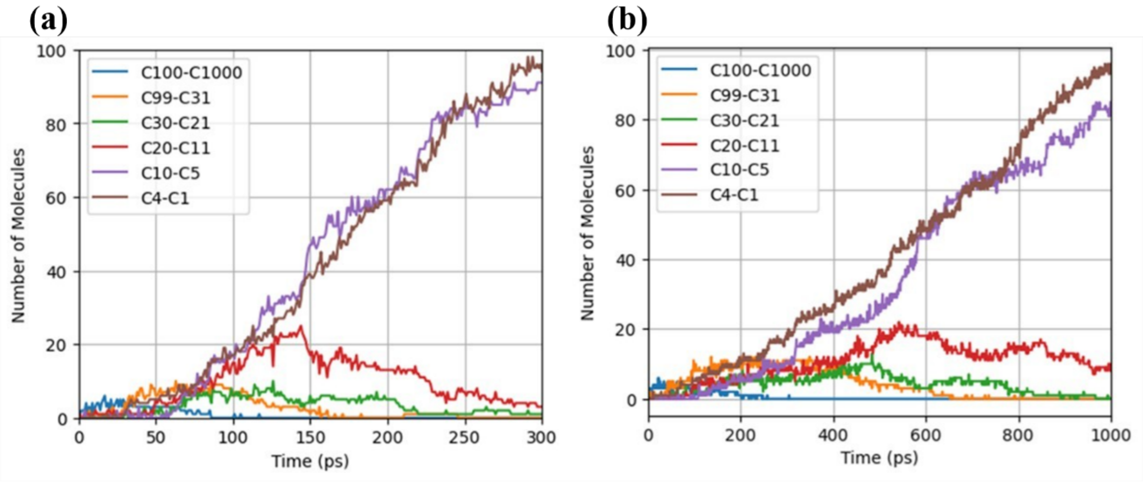

Figure S2 Time evolution of molecular species during PET hydrolysis at 1800 K under two densities: **(a)** 0.99 g/cm³ (average pressure: 2.66 GPa) and **(b)** 0.81 g/cm³ (average pressure: 1.44 GPa), showing the fragmentation of PET into chains of varying lengths.

For further investigation, MD simulations were conducted with different pressures (**Figure S2**) through changes in system density. At 1800 K, the simulated conditions included densities of 0.99 g/cm³ (average pressure: 2.66 GPa) and 0.81 g/cm³ (average pressure: 1.44 GPa). While the pressures in these simulations are significantly higher than those in the experiments due to the elevated temperature, this deliberate pressure gap enables us to assess the consistency of trends across different conditions. Despite differences in reaction rates between the two densities, the trends in species evolution and fragmentation were identical, with larger fragments persisting longer at higher densities and smaller species forming more rapidly at lower densities.

3. References

(1) Pereira, P.; Savage, P. E.; Pester, C. W. Neutral Hydrolysis of Post-Consumer Poly(ethylene terephthalate) Waste in Different Phases. *ACS Sustain. Chem. Eng.* **2023**, *11* (18), 7203–7209. https://doi.org/10.1021/acssuschemeng.3c00946

(2) Mishra, S.; Zope, V. S.; Goje, A. S. Kinetics and Thermodynamics of Hydrolytic Depolymerization of Poly(ethylene terephthalate) at High Pressure and Temperature. *J. Appl. Polym. Sci.* **2003**, *90* (12), 3305–3309. https://doi.org/10.1002/app.13065
